# Donors and Recipients of Living Kidney Donation: A Qualitative Metasummary of Their Experiences

**DOI:** 10.1155/2011/626501

**Published:** 2011-06-21

**Authors:** Deborah Ummel, Marie Achille, Jessina Mekkelholt

**Affiliations:** Department of Psychology, Université de Montréal, P.O. Box 6128, Downtown Station, Montreal, QC, Canada H3C 3J7

## Abstract

With the notable growth in the qualitative investigation of living kidney donation, there is value in aggregating results from this body of research to learn from accumulated experience. The present paper aims to draw a complete portrait of living donors' and recipients' experience of donation by metasummarizing published studies. We found that donors' experience, particularly the decision-making process, has been more extensively studied than the recipients' perspective. Donors differ in their initial level of motivation to donate but on the whole report positive experiences and personal benefits. They also identify difficult periods and the need for additional resources. Recipients report an often positive but more ambivalent reaction to donation. In terms of relational issues between dyads, while the topic remains understudied, the donor-recipient relationship and gift reciprocity have received the most attention. Results are discussed in terms of their implications for future practice and research.

## 1. Introduction

Renal transplantation is considered the best treatment in the case of end-stage renal disease [1], as it is associated with better quality of life and a longer life expectancy [2] and is more profitable economically [3] than dialysis. Given the shortage of renal graft from deceased persons [4, 5], the low risk involved for the donor [6, 7] and the improved quality of life likely to result for the recipient [8, 9], living kidney donation is currently being promoted and increasingly practiced in all western societies [10].

Living kidney donors have received much research attention for evident ethical reasons [11], namely, to establish the low risk involved and positive long-term effects of living donation. Numerous quantitative studies conclude that donors usually experience their donation act in a positive manner and that they would reiterate their gesture if possible [12–18]. In terms of their quality of life, donors' scores are higher than reference populations, even after surgery [13, 19]. Donors also report personal benefits from their donation experience, such as a higher self-esteem or well-being after donating [14, 20–22]. Donors report personal growth (e.g., an increased appreciation for the value of their own life), interpersonal benefits (e.g., an increased respect and admiration by family and friends), and even spiritual benefits (e.g., the donation is seen as a way of honoring a higher spiritual being) [20–22]. In spite of this overall positive picture, it is important to mention that a small proportion of donors report poor experiences with donation [13], especially when the renal graft did not function as expected for the recipient [16]. Cases of depression, adjustment disorder, and anxiety have been reported, even when surgery outcomes were positive for the recipient and without any medical complication for the donor [19, 23].

While quantitative studies give a very valuable but often partial description of a complex process such as living donation, qualitative studies, typically conducted on a smaller scale, allow a complementary in-depth exploration of complex human experiences [24, 25]. As mentioned previously [26], if living kidney donors have been brightly studied, there is a lack of studies addressing the particular situation of receiving a kidney from a live donor. In the present study, we are aggregating results pertaining to the experience of both donors and recipients of a living kidney donation in order to offer a complete picture of the donation process as it has been examined thus far in the empirical literature. Summarizing these qualitative results will add to the transplantation community's continuing clinical and research efforts to understand the accumulated experience of living donation. This seems particularly timely in the current context of the active promotion of living donation and access to novel donation avenues (e.g., paired exchange).

The present paper focuses uniquely on living kidney donation, as other forms of living donations (e.g., liver, partial lung) are performed in very different contexts in terms of the urgency with which decisions have to be made, the risks involved for the donors, and the limited alternative options available to intended recipients.

## 2. Materials and Methods

### 2.1. Selection Criteria

We included qualitative studies that used interviews or focus groups to explore donors' and recipients' experience of living kidney donation. We included solely studies published in peer-reviewed journals and written in English, French or German, so that we could understand them completely. We excluded studies that reported only quantitative data or used structured questionnaire as their only method for data collection.

### 2.2. Article Retrieval

In November 2010, we conducted a literature search in three databases: PsycINFO (1987 to November Week 1 2010), CINAHL and Medline (1996 to October Week 4 2010). In PsycINFO, we obtained 75 results with the use of the keyword “living don*” (*denotes truncation), in CINAHL we obtained 100 results by using “living don* AND renal or kidney” and in Medline, we obtained 79 results combining the following keywords: “living don* AND kidney or renal AND qualitative or focus group* or interview* or case stud*”. After removing articles that appeared in more than one database, we ended up with 236 articles and examined their title, abstract and eventually the entire study to select those meeting our selection criteria. To confirm no omission of relevant articles, we scrutinized reference lists of literature reviews [27, 28] and of the 15 articles selected for this review (see Table 1).

### 2.3. Synthesis of Findings

The 15 articles were metasummarized following techniques described by Sandelowski and Barroso [44, 45]. The articles were reviewed and relevant findings were extracted from each study included in the review. We then grouped the findings in common topical domains and summarized them into abstracted findings (Table 2) [46]. Subsequently, we calculated *frequency effect sizes of findings* and *intensity effect sizes of studies*, considering each study as one unit of analysis and weighting each study equally [44, 47]. The intensity effect size of studies was calculated by dividing the number of findings of each study by 54, the total number of finding extracted through our metasummary. The frequency effect size of findings was calculated by dividing the number of studies mentioning a particular finding by 15, the total number of studies included in our metasummary. The synthesis of findings is shown in Table 2, with a frequency effect size reported for each finding (e.g., altruistic and natural decision's frequency effect size is 46.7% because this finding appeared in 7 of 15 studies) and an intensity effect size reported for each study (e.g., [41] has a 33.3% intensity effect size, because it contains 18 findings out of total 54 reported in the present metasummary).

## 3. Results

**Figure 1:**
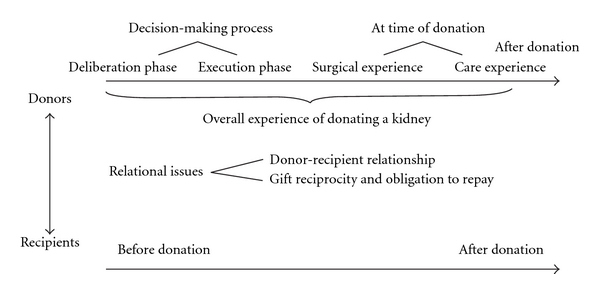
Summary of the major themes of our metasummary.

Results are presented following the typical chronology of the living kidney donation process, namely, results pertaining to the decision-making phase are presented first, followed by those pertaining to the timing of donation, then by those relevant to the period after donation. We begin with the donors' experience, as it has been more extensively investigated in the current literature. We next present the literature on recipients, and finally address relational issues between donors and recipients. A schematic representation of the results is provided in Figure 1. Figure 1 also illustrates that donor issues have been studied more in depth than relational or recipients' issues. Detailed results are presented in Table 2.

### 3.1. Donors

#### 3.1.1. Decision-Making Process

The donors' decision-making process usually starts with a deliberation phase where donors begin having thoughts about giving a kidney to a recipient. This typically happens before the decision to be tested for compatibility [29]. Donors' decision-making process appears to be influenced by several factors that differ from one donor to the other. Awareness of recipient's suffering appeared to be a consensual and powerful motivation and an influential factor in donors' decision [32, 33, 36–38, 40–42]. For some donors, it was an altruistic and natural decision meant to improve the recipient's health and quality of life [29, 32, 34, 35, 37, 41, 42] but this decision could also be more philosophical or spiritual in nature [29, 34, 42]. Some studies described donors' decision as carefully thought through [29, 34–36, 40, 42], whereas other stated it was a quick and straightforward decision [36, 41]. In addition, the decision was also often described as sufficiently informed and rational [32, 36, 40, 41].

Numerous studies highlight familial issues, but no clear consensus from these different studies emerged in terms of how certain types of relationships (e.g., siblings, parent-child) impacted decision-making or outcome. Within families where more than one potential donor was available, there was often mediation and negotiation in order to find the best family member to assume this role [38]. It seems that the reason expressed to become a donor could depend on the familial relationship status with the recipient (e.g., being a mother or a brother), but the findings extracted lead to no consensus on this [29, 31, 32, 34, 35, 38, 41, 42]. However, one consensus was found around the absence of pressure from others donors felt in their decision-making process [32, 35, 36, 41]. One study argued that intimacy with the recipient is an important factor in the decision-making process, and the more intimate the donor and the recipient are, the higher the wish to give [29]. Even when wishing to give a kidney, donors often felt anxiety during the process [34, 36], from the risk of surgery [42] or the stress of being declined as a donor [40] for example.

The timing at which donors made their final decision differed greatly between participants and studies. Timing partly depended again on the familial relationship with the recipient, but not solely [31, 36]. One study reported that the medical examination was experienced as a difficult stage, the worst step, because it was long and involved stress over delays and anxiety regarding results [36]. Being reminded of the possibility to withdraw was reported as experienced negatively by participants in two studies. Indeed, after having made the decision to donate, donors found it unimaginable not to proceed [40] and they understood the repeated information that they could withdraw as a doubt about their decision that had to be defended and maintained [36].

Social support has been described as important during the decision-making process even though results were not unanimous on that subject. Some donors appreciated the support received from family, friends, colleagues and the broader community who endorsed their decision [34, 42]. However, in other cases, members of the immediate family were not considered suitable supporters as they were reported as anxious about the surgery [36]. More generally, donors expressed that there is a need for more emotional support, as existential interrogations, such as questions about life and death and the meaning of life, were activated during the donation process [36].

One single study [29] detailed the execution phase, being the phase where donors finally arrived at their decision. This study proposed a typology of different donor types: the voluntary type, the compromising type and the passive type. Donors of the voluntary type have an intense will to give, their decision-making process is straightforward and they have strong intimacy with their recipient. They are so determined to give that the process of compatibility testing can be stressful because of the fear of being rejected as a donor. On the other hand, donors of the compromising type have a moderate will to give, resulting in a more complicated decision-making process and a passive participation in compatibility tests. These donors volunteer when the test results are positive, feeling that they have no other choice. In addition, most donors of this type receive financial compensation. Donors of the passive type have a low will to give and are reluctant to take compatibility tests. Their intimacy with the recipient is the lowest. All passive donors are persuaded by family members with financial compensation.

#### 3.1.2. At the Time of Donation

The surgical experience of donation was a theme often examined in the articles reviewed. Just before surgery, donors varied in their attitudes regarding surgery. Although some approached it in a calm manner, it was an anxiety provoking event for others, leading authors to suggest that each donor's needs in this period are unique [40]. Some donors made preparations in case they were to die, such as writing a will [36] and the emotional component of their experience increased in the days leading to the operation [34]. After surgery, numerous studies reported that donors had experienced pain [35, 36, 40, 41, 43], nausea [35, 41], exhaustion [36, 38, 39, 43] and scar problems [41]. These effects were expressed as more important than expected, and at some points donors felt they had not been well prepared for these effects. Psychological strain was also mentioned in several studies [33, 36, 39, 40, 43]. Insufficient pain relief could lead to psychological symptoms and reduced emotional capacity in some donors [33, 36, 38, 39], they could experience a sense of loss or grief after donation [42] and the fear of rejection was also an important concern [43]. Regarding the care experience, several studies reported positive experiences [32, 34, 36, 40], such as care that was trustworthy and honest [40], namely, a call from a coordinator some weeks after discharge [36] and the availability of support from the transplant health care team when needed [34]. There were some negative aspects to their experiences as well, such as a lack of information after discharge [40] and a distressing and uncomfortable experience at the hospital [38]. Some donors also felt abandoned and ignored by the staff [36]. One study highlighted that donors would appreciate receiving better psychological care in such critical situations as regressive reactions, pain attacks, and transplant rejection episodes [36].

#### 3.1.3. After Donation

Regarding medical followup postdonation, one study reported that the majority of donors expressed satisfaction regarding the care received, but that some donors expressed frustration due to unmet expectations from health professionals [39]. With their care mostly being left in the hands of their family doctors, another study reported that donors would have welcomed additional contact with the transplantation health care team [40]. Donors were reported as having few worries regarding their future health with only one kidney [33, 35]. One study stated that since donation, donors had become proponents of living kidney donation in the community [42]. Advices and recommendations to future donors were also discovered. In one study, donors stressed for future donors the importance for them to make the decision personally and free from any pressure [42]. Donors in a separate study emphasized that future donors need to be determined and should not start hesitating [36]. A large consensus was found regarding the absence of regret among donors, and the fact that they report they would make the same decision again [31, 33, 37, 38, 41, 42]. About one year after donation, all donors were generally physically back to normal, in the sense that they had a good recovery and did not feel any different physically [39, 41, 43].

#### 3.1.4. Overall Experience of Donating a Kidney

In studies looking at the overall donation process, the experience of living kidney donation has been described as complex, multi-faceted, and as including physical, mental and interpersonal challenges [34, 35]. One large consensus found among studies was that donors were reported as having experienced benefits [32] such as personal growth [35], increased self-esteem [42], a sense of accomplishment and pride [33, 37, 39], immense personal satisfaction [41] and a change in their outlook on life [43]. The donation was also considered a meaningful action, in the sense of having contributed to a better life for another person [35, 39, 42]. However, being a donor was also described as an unfamiliar trajectory as it implied for a fit person to be surgically traumatized [35] and it also led to conflicting roles, as the donor was simultaneously a patient, a close relative to the recipient, and a family member, which could lead to a stressful convalescence [35, 38–40]. When transplantation failed, the experience was reported as unexpected and distressing, and donors' responses were described as depression and sorrow, a feeling of emptiness and a loss of strength [32, 33, 39]. Another study highlighted that there is a particular need for followup after discharge when the graft fails [40].

### 3.2. Recipients

#### 3.2.1. Before Donation

In order to get a kidney from a live donor, some recipients asked the donor directly, whereas others preferred to wait for the donor to volunteer. One study reported that recipients had different ways of asking for a kidney. Some recipients preferred to ask face to face, whereas others thought that writing a letter or an email gave the donor the option to think about it before making the decision [37]. On the contrary, other recipients were unwilling to introduce the topic, wanting the donor to volunteer and, therefore, had not asked any potential donor [37, 41]. Some recipients felt anxiety about the risks to the donor's health and well-being [30, 31, 41] and a few had misgivings about accepting the offer [31]. Other recipients were afraid the donor was just being polite by undergoing compatibility tests [37]. In accepting the kidney, intended recipients often asked themselves whether or not they would do the same and donate a kidney to another individual [38], and some believed it would be insulting to the donor-to-be to refuse their extraordinary offer [30]. In addition, some recipients found it fair to accept given they had been ill for a long time [30], some expressed positive feelings with regard to the decision to undergo transplantation [33], and some stated that having a close relationship with the donor was important [37].

#### 3.2.2. After Donation

Recipients were found to be extremely grateful to the donors for their donation. They all thanked them for their gesture, but many found it uneasy to articulate their gratitude fully [31, 41]. Most recipients had no regrets about transplantation, however adolescent recipients expressed some regrets largely because of the perceived obligation to accept a kidney proposed by a family member [31]. The transplantation's impact on the recipient's health was reported as significant both for the recipient's life and for his or her family [41]. However, some recipients felt psychological strain, such as depressive symptoms or anxiety, and this was reported to happen despite a favorable medical outcome [33] and others lived the overall donation experience negatively [33].

### 3.3. Relational Issues

#### 3.3.1. Donor-Recipient Relationship

Numerous studies reported that after living kidney donation, the relationship between the donor and the recipient remained the same (e.g., close) or sometimes even improved [30, 33, 35, 39, 41, 42]. However, there were also cases were the relationship deteriorated [31, 33]. It seems that familial issues played a role in the evolution of the relationship, but there was no consensus in the two studies that mentioned this [30, 31]. Finally, donors and recipients were also found not to have any profound discussion about the transplantation 10 months after transplantation. They felt that even if the transplantation changed their lives, they needed to move on to something else [41].

#### 3.3.2. Gift Reciprocity and Obligation to Repay

Several studies reported that donors had no expectation regarding repayment or gratitude from the recipient [31, 35, 41]. However, some recipients perceived an obligation, such as always being grateful [31, 38] and becoming extremely cautious about their own health in the fear they would be held responsible in case of rejection [38, 42]. Some recipients gave a gift to thank their donor, for example through a small ritual on the anniversary of the transplant date [30, 42]. The recipient's sense of gratitude had the potential to alter the relationship [33, 41] and it was reported to be sometimes difficult to cope with having received the gift of donation [30]. Some recipients reduced their feeling of indebtedness by stressing that the donor had also gained from the donation or by playing down the significance of the gift [30].

## 4. Discussion

The present paper aimed to aggregate results pertaining to the experience of both donors and recipients of a living kidney donation in order to offer a complete picture of the donation process.

With respect to the donors' perspective, the decision-making process has been most extensively studied and constitutes the most deeply detailed and complete theme of our metasummary. Considering the implications of a live donation-namely, experiencing extensive tests, undergoing a surgical intervention selflessly and losing one kidney-the decision-making process is crucial and it is common sense that it was one of the first aspects to be studied [29]. In addition, the literature highlights that awareness of recipient's suffering constitutes a consensual motivation to donate as this finding appeared in 53.3% of studies reviewed, meaning that this finding was found in the majority of studies. The degree of intimacy in the relationship with the intended recipient better predicts the decision to donate than simply the type of relationship (e.g., parent, sibling, etc.). A decision described as altruistic, seen as natural and meant to improve recipient's health also had a very high-frequency effect size as this finding emerged in 46.7% of the studies reviewed. This type of decision is also seen in many clinical situations.

Our metasummary further highlights the overall experience of donors, who report having no regret. It is worth noting that the two findings “donors would reiterate their gesture” and “having personally benefited from this process” are findings with very high-frequency effect sizes. Forty percent of the reviewed studies indicate that donors would reiterate their gesture and 53.3% of them report personal benefits for donors. This is also consistent with results from quantitative studies previously cited [12–18], and thus strengthens this common aspect of donors' experience.

Reviewed studies, however, also confirm there are challenging aspects to the donation process. Surgical effects were often more important than expected for donors, and some felt they had not been adequately prepared. Experience of pain, nausea and exhaustion were reported among 46.7% of the reviewed studies. The overall trajectory of donation was described as an experience unlike any other and somewhat unfamiliar; the multiple roles it involved were sometimes a source of strain. In addition, when transplantation outcomes were negative for the recipient, there was an increased risk of emotional and psychological difficulties for donors.

For recipients of a live donation, the experience had many positive aspects but also involved ambivalence to the situation. Candidates for transplantation vary greatly in their willingness to ask their family and friends for a kidney or even introduce the topic. When a kidney is offered, acceptance is preceded by a reflexive process that is concluded with some form of justification for accepting, which is different for each recipient. After donation, recipients experience significant health improvement and are on the whole very grateful to their donor. There is, however, a risk for psychological strain in the context of certain types of relationships between donor and recipient or due to the constraints of the transplantation process (e.g., medical adherence posttransplantation).

In terms or relational issues, our metasummary highlights that the donor-recipient relationship often remains the same, improves or becomes closer, a finding extracted in 40% of studies reviewed. There is, however, also evidence of a risk of deterioration in cases of conflict between donor and recipient, problems and strain related to the transplantation or a relationship already difficult before the transplantation, a finding which was only found among 13.3% of studies reviewed. The issue of gift reciprocity and obligation to repay was also mentioned as having the potential to alter the relationship.

These results suggest avenues to strengthen clinical practice. However, we recognize that practices can likely vary across centers due, in part, to varying degrees of professionals' experience with live donation and availability of resources. Improvements suggested by donors include better preparation for the postsurgical period, easily accessible psychological support throughout the process but also during this particular period, and continued followup by the transplantation health care team following donation. Access to psychological support has also been advocated in prior studies [40]. In light of donors' discourse on personal benefits of donation and active advocacy following donation, these aspects are important to acknowledge, and should also be shared with potential donors and intended recipients at the outset of the process. Indeed, ethical decision-making involves informing donors about all risks and complications that may occur, but also about potential benefits of the transplant for both recipients and donors.

For recipients, one of the most sensitive and challenging aspects remains informing others about the possibility of donating and the advantages of living kidney donation. This is where transplantation health care teams may be called upon to play a more active role in informing the community of potential donors about this option. How and to whom this publicity may be directed, however, is to be discussed within the boundaries of professional and ethical responsibility. After donation, recipients' discourse suggests a need for increased attention to possible psychological strain, and how to optimize coping with issues of gratitude and reciprocity.

Implications for research are many. For one, recipients' experience of a live kidney donation has received little research attention. Indeed, only one single study explored recipients' experience [30] and five studies out of 15 addressed some aspects of it [31, 33, 37, 38, 41]. In comparison, the donors' perspective was the focus of nine out of 15 studies [29, 32, 34–36, 39, 40, 42, 43]. In light of this and of available results suggesting that experience of receiving a kidney from a live donor is complex and different from the experience of receiving a kidney from a deceased donor, additional research is needed to investigate the perspective of recipients and donor-recipient dyads. Relational issues in the context of living donation and after transplantation also have received little research attention and, in order to be able to intervene adequately with donors and recipients experiencing relation problems, there is a need to further investigate this area.

It is interesting to note that studies included in the present metasummary emerged from diverse social and cultural contexts, ranging from known-to-be egalitarian societies such as Sweden [30, 32, 36] to highly hierarchical ones such as Korea [29]. In this regard, caution is advised in drawing early conclusions on the basis of our metasummary. Also, given the diversity in the findings emerging from these different contexts, there is ground to explore in more detail the impact of social and cultural factors particularly on the decision-making process and on the psychosocial outcomes of transplantation involving live donation.

Although achieved rigorously and systematically, this metasummary has several limitations. First, we restricted our searches to peer-reviewed journals published in English, French and German, thus eliminating the possibility to include research conducted in theses and dissertations. Secondly, studies retrieved focused on different issues and groups, varying from donors only, to donors and recipients, to recipients only. Even if this highlights the fact that some aspects are still understudied (e.g., the recipients' experience), this could potentially lead to a snap judgement. However, this work offers a complete, empirically-documented overview of donors' and recipients' experience of the donation process.

## 5. Conclusions

A major strength of this work is to offer a complete picture of donors' and recipients' experience of the donation process based on empirical published literature with a rigorous and systematical metasummary technique. These results could be especially useful for new professionals working in the living kidney transplantation field, as well as professionals intervening solely at one particular step of the process. Health care professionals can also gain a certain knowledge about their impact in the process. At a time where there is an active promotion of living kidney donation and access to novel donation avenues, such as paired exchange, it is particularly important to have a better understanding of donors' and recipients' experience of this process.

## Figures and Tables

**Table 1:** Characteristics of studies included in the metasummary.

Authors	Year	Reference	Research design/methodology	Sample	Study purpose/research question as reported by authors	Country
Yi	2003	[29]	Grounded theory using semistructured interviews	14 living donors	Explore what people experienced when deciding to donate a kidney and explore associated issues and concerned when they made their decisions	Korea

Sanner	2003	[30]	Follow-up interviews 3, 6, 12, and 24 months posttransplantation	12 heart recipients, 12 living-kidney recipients and 11 necro-kidney recipients	To examine how organ recipients in late modernity conceived the special features that distinguish the transplantation from other treatments, namely, that vital, “living”*‌* organ are transferred from one human being (deceased or alive) to another	Sweden

Franklin et al.	2003	[31]	Retrospective semistructured interviews between 1 and 5 years after transplantation (phenomenological approach)	50 donors and partners and recipients and partners (study A)	Not reported	United Kingdom

Haljamäe	2003	[32]	Qualitative interviews (phenomenographic approach)	10 living donors	Assess and describe the remaining experiences of donors more than 3 years after early recipient graft loss or death of the recipient	Sweden

Heck et al.	2004	[33]	Case studies by catamnestic interviews with donors and recipients	31 donor-recipient dyad	Examine the psychosocial effects of living donor kidney transplantation for donors and recipients under successful as well as complicated circumstances	Germany

Walsh	2004	[34]	Semi-structured interview (interpretative phenomenological analysis)	8 living related donors	Explore psychological experience, motivation, and meaning associated with decision-making processes	Ireland

Andersen et al.	2005	[35]	Individual in depth interviews (empirical phenomenological method)	12 living donors	Explore physical and psychosocial issues related to the experiences of living kidney donors 1 wk after open donor nephrectomy	Norway

Sanner	2005	[36]	Interviews the day before nephrectomy and 3 weeks afterwards	39 living donors	Explore the donation process of a heterogeneous group of genetically and nongenetically related living kidney donors	Sweden

Waterman et al.	2006	[37]	Focus group methodology	26 recipients, 4 donors and 3 family members	Understand the psychological barriers and educational needs of potential recipients regarding living donation	United States

Crombie and Franklin	2006	[38]	Ethnographic interviews	50 donors, recipients and nondonors	Explore the family experience of live donation from psychological, social and cultural perspectives	United Kingdom

Andersen et al.	2007	[39]	Follow-up interviews (phenomenological approach)	12 living donors	Explore experiences regarding physical and psychosocial health during the first year after donor surgery	Norway

Brown et al.	2008	[40]	Qualitative interviews (phenomenological approach)	12 living donors	Explore living kidney donors' perceived experiences with the health care system from the period prior to being tested as a potential donor, through to postdonation discharge and followup	Canada

Gill and Lowes	2008	[41]	Interviews (phenomenological approach)	11 donor-recipient pairs	To explore the experience of donors and recipients throughout the live transplantation process and the relevance of the theory of “gift exchange” as a framework for exploring and understand donors and recipients experiences of live transplantation	England

Brown et al.	2008	[42]	Semi-structured interviews (phenomenological approach)	12 living donors	Gain a deeper understanding of the decision-making processes and psychosocial issues for living kidney donors	Canada

Williams et al.	2009	[43]	Grounded theory using interviews	18 living donors	Explore and describe the experiences of persons who had donated a kidney within Western Australia	Australia

**Table 2:** Synthesis of findings with frequency effect size of each finding (how often a particular finding appeared in the body of literature reviewed) and intensity effect size of each study (how much each study contributes, in terms of the number of findings it includes, to the overall body of literature reviewed).

Finding		Frequency Effect Size of Findings (%) ↓	Yi, 2003 [29]	Sanner, 2003 [30]	Franklin et al., 2003 [31]	Haljamäe, 2003 [32]	Heck et al., 2004 [33]	Walsh, 2004 [34]	Andersen et al., 2005 [35]	Sanner, 2005 [36]	Waterman et al., 2006 [37]	Crombie and Franklin, 2006 [38]	Andersen et al., 2007 [39]	Brown et al., 2008 [40]	Gill and Lowes, 2008 [41]	Brown et al., 2008 [42]	Williams et al., 2009 [43]

	Intensity Effect Size of Studies (%) →		14.8	11.1	20.4	14.8	22.2	16.7	24.1	31.5	13.0	18.5	16.7	22.2	33.3	29.6	7.4
Donors	*Decision-making process *																
	*Deliberation phase *																
	Awareness of suffering	53.3				•	•			•	•	•		•	•	•	
	Altruistic and natural decision	46.7	•			•		•	•		•				•	•	
	Spiritual—philosophical decision	20.0	•					•								•	
	Carefully thought through decision	40.0	•					•	•	•				•		•	
	Quick and straightforward decision	13.3								•					•		
	Informative decision	26.7				•				•				•	•		
	Familial issues	53.3	•		•	•		•	•			•			•	•	
	No pressure	26.7				•			•	•					•		
	Intimacy with recipient	6.7	•														
	Threat—anxiety	26.7						•		•				•		•	
	Time of decision	13.3			•					•							
	Examinations: difficult stage	6.7								•							
	Withdraw possibility	13.3								•				•			
	Social support	20.0						•		•						•	
	*Execution phase *																
	Voluntary type	6.7	•														
	Compromising type	6.7	•														
	Passive type	6.7	•														
	*At time of donation *																
	*Surgical experience *																
	Just before surgery	20.0						•		•				•			
	Pain, nausea, exhaustion, scar	46.7							•	•		•	•	•	•		•
	Psychological strain	40.0					•			•		•	•			•	•
	*Care experience *																
	Positive experience	26.7				•		•		•				•			
	Negative experience	20.0								•		•		•			
	Wish for better psychological care	6.7								•							
	*After donation *																
	Medical followup	13.3											•	•			
	Concerns regarding future health	13.3					•		•								
	Living donation active promotion	6.7														•	
	Advices for others	13.3								•						•	
	Same decision again—no regrets	40.0			•		•				•	•			•	•	
	Back to normal	20.0											•		•		•
	*Overall experience of donating a kidney *																
	Complexity	13.3						•	•								
	Benefits for donors	53.3				•	•		•		•		•		•	•	•
	Donation: meaningful action	20.0							•				•			•	
	Being donor: unfamiliar trajectory	6.7							•								
	Conflicting donor roles	26.7							•			•	•	•			
	When transplantation fails	26.7				•	•						•	•			

Recipients	*Before donation *																
	Different ways of asking for a kidney	6.7									•						
	Wait for donors to volunteer	13.3									•				•		
	Accepting a kidney from a live donor	40.0		•	•		•				•	•			•		
	*After donation *																
	Being grateful to the donor	13.3			•										•		
	No regrets	6.7			•												
	Regrets	6.7			•												
	Benefits for recipients' health	6.7													•		
	Psychological strain	6.7					•										
	Negative experience	6.7					•										

Relational Issues	*Donor-recipient relationship *																
	Close, stable and possible improvement	40.0		•			•		•				•		•	•	
	Conflicts and deterioration	13.3			•		•										
	Familial aspects	13.3		•	•												
	Need to move on with their lives	6.7													•		
	*Gift reciprocity and obligation to repay *																
	No expectations from donors	20.0			•				•						•		
	Recipients' obligation to be grateful	13.3			•							•					
	Recipients' obligation regarding the graft	13.3										•				•	
	Recipients' gift to the donor	13.3		•												•	
	Recipients' gratitude	20.0		•			•								•		
	Way to reduce recipients' debt	6.7		•													

•: presence of a given finding.
